# Compared Phenolic Compound Contents of 22 Commercial Fruit and Vegetable Juices: Relationship to Ex-Vivo Vascular Reactivity and Potential In Vivo Projection

**DOI:** 10.3390/antiox9020092

**Published:** 2020-01-22

**Authors:** Alexis Matute, Jessica Tabart, Jean-Paul Cheramy-Bien, Bernard Pirotte, Claire Kevers, Cyril Auger, Valérie Schini-Kerth, Jacques Dommes, Jean-Olivier Defraigne, Joël Pincemail

**Affiliations:** 1 Laboratory of Plant Molecular Biology and Biotechnology, UR InBios-Phytosystems, University of Liège, Sart Tilman, 4000 Liège, Belgium; afmmatute@student.uliege.be (A.M.); jessica.tabart@alumni.uliege.be (J.T.); c.kevers@uliege.be (C.K.); j.dommes@uliege.be (J.D.); 2 Department of Cardiovascular Surgery, CREDEC and Plateform Nutrition Antioxydante et Santé, CHU and University of Liège, Sart Tilman, 4000 Liège, Belgium; jp.cheramy-bien@chuliege.be (J.-P.C.-B.); jo.defraigne@chuliege.be (J.-O.D.); 3 Laboratory of Medicinal Chemistry, Center for Interdisciplinary Research on Medicines (CIRM), Sart Tilman, 4000 Liege, Belgium; b.pirotte@uliege.be; 4 Laboratory of Biophotonic and Pharmacy, Faculty of Pharmacy, University of Strasbourg, 67401 Illkirch, France; cyril.auger@unistra.fr (C.A.); valerie.schini-kerth@unistra.fr (V.S.-K.)

**Keywords:** fruit and vegetable juices, phenolic compounds, vasorelaxation effect, rat aorta

## Abstract

The real impact of polyphenol-rich vegetable and fruit juice intake on cardiovascular health remains a matter of controversy. In the present study, rat aorta segments immersed in an organ bath (OB) were used to explore whether the total polyphenol content and/or individual phenolic compound contents of 22 commercial vegetable (*n* = 3) and fruit juices [(citrus (*n* = 5), berries (*n* = 10), apple (*n* = 2), pineapple (*n* = 2)] might be associated with vascular tone. Red juices (particularly blackcurrant) and lemon juice caused the most marked vasorelaxation, its amplitude being endothelium dependent or not according to the volume ratio of juice to initial OB solution V_juice_/V_OBS_). At volume ratios 5% and 10%, both the juice and OB total polyphenol for all juices and total anthocyanin contents for berry juices significantly correlated with aorta vasorelaxation intensity. This was not the case for total or individual flavonols (except kaempferol) or for total or individual flavanols (except epigallocatechin gallate). If one relates our measured concentrations of individual phenolic compounds in OB to what is known about their physiological concentrations, and given our evidenced correlations between compound concentrations and vasorelaxation intensity, kaempferol, epigallocatechin gallate and peonidin-3-*O*-glucoside seem to emerge as the interesting phenolic compounds likely to be responsible for the potent vasorelaxation observed with fruit juices, and more particularly blackcurrant ones. Clinical investigation is required, however, to confirm our observations.

## 1. Introduction

Long considered anecdotal, the role played by antioxidants, and particularly polyphenols, is now viewed as pivotal in human disease prevention [1,2,3,4,5]. According to their chemical structure, natural polyphenols can be divided into lignans, lignins, stilbenes, styrylpyrones, arylpyrones, coumarins, tannins, phenolic acids (i.e., benzoic and cinnamic acid derivatives), and flavonoids (i.e., flavonols, flavanones, flavones, flavanols or catechins, anthocyans, and isoflavones). Flavonoids are particularly abundant in fruits, vegetables, and plant-derived products such as fruit juices, dark chocolate, green tea, red wine, coffee, and olive oil. Zutphen’s study [6] was the first to evidence, in an elderly Dutch population, a strong inverse correlation between flavonoid consumption and the risk of developing cardiovascular disease. Using a food frequency questionnaire and the Phenol-Explorer database, Mendoça et al. [7] recently described the same relationship in a prospective cohort of 17,064 Spanish middle-aged university graduates (the “Seguimiento Universidad de Navarra” or SUN study). After adjusting for potential confounders, participants with a higher flavonoid intake (fifth quintile) showed a 47% lower incidence of cardiovascular events than those in the lowest quintile.

Polyphenols exert various actions likely to explain their cardioprotective effect. They notably have platelet aggregation and anti-inflammatory properties as well as ability to increase the high density lipoprotein (HDL)/low density lipoprotein (LDL) cholesterol ratio. They can also act as direct free radical scavengers but only at the stomach level. Despite low oral bioavailability and rapid metabolism, polyphenols may, however, at cellular level produce—through moderate auto-oxidation—small amounts of reactive oxygen species (ROS), which induces an antioxidant adaptive response in cells through the activation of the Keap1/Nrf2/ARE pathway. This results to over-expression of genes coding for antioxidant enzymes (Antioxidant Response Element) [8]. The physiological mechanism most often put forward to explain cardiovascular protection due to polyphenols is their ability to regulate arterial blood pressure by maintaining or improving endothelial function [9,10]. Indeed, polyphenols can increase the bioavailability of nitric oxide (NO), which has potent vasodilator properties. Many papers have evidenced an inverse relationship between endothelial dysfunction, as evaluated on the basis of flow-mediated dilation (FMD), and the risk of developing cardiovascular disease [11,12,13]. In contrast, important meta-analyses have clearly shown that a Mediterranean diet [14] or regular consumption of fruits, vegetables, or plant-derived products (dark chocolate, green tea, olive oil) can improve endothelium function and hence reduce both systolic and diastolic arterial pressure [15].

The aim of the present study was to examine the ability of several popular phenolic-compound-containing fruit and vegetable juices to improve ex-vivo endothelial function. Another objective was to identify the most effective subclasses of polyphenols and to see more generally whether a vasodilator effect could be observed in the presence of polyphenols at physiological concentrations (µM) [16].

## 2. Materials and Methods 

### 2.1. Materials

The following reagents were purchased from Merck KGaA, Darmstadt, Germany: cyanidin-3-*O*-glucoside (CyG), catechin (C), picatechin (EC), epicatechin gallate (ECG), gallocatechin (GC), epigallocatechin (EGC), and epigallocatechin gallate (EGCG). Cyanidin-3-*O*-rutinoside (CyR), delpinidin-3-*O*-glucoside (DG), delphinidin-3-*O*-rutoside (DR), peonidin-3-*O*-glucoside (PG), malvidin-3-*O*-glucoside (MG), delphinidin (D), cyanidin (Cy), petunidin (Pet), pelargonidin (Pel), peonidin (Peo), malvidin (M), kaempferol, myricetin, and quercetin were obtained from Extrasynthese Lyon, France.

Further, twenty-two commercial juices were bought in Belgian, French and German supermarkets and classified as vegetable or fruit juices. These were: (1) Tomato (*Carrefour*), (2) Tomato (*Biotta*), (3) Carrot (*Biotta*), (4) Orange (*Carrefour*), (5) Pure Orange (*Vitamont*), (6) Lemon (*Bonneterre*), (7) Grapefruit (*Carrefour*), (8) Pure Grapefruit (*Vitamont*), (9) Grape (*Materne*), (10) Pure Grape (*Vitamont*), (11) Pomegranate (*Biotta*), (12) Blackcurrant (*Biotta*), (13) Blackcurrant (*Natreen*), (14) Blackcurrant (*Jacoby bio*), (15) Blackcurrant (*Van Nahmen*), (16) Blackcurrant (*Schlör Nectar*), (17) Blackcurrant (*Gut and Gunstig*), (18) Blackcurrant (*Jacoby*), (19) Pineapple (*Carrefour*), (20) Pineapple Juice (*De Drie Wilgen*), (21) Apple *(Carrefour*), and (22) Pure Apple (*Vitamont*) (Figure 2). All beverages were 100% pure vegetable or fruit juices except the blackcurrant ones that were nectar.

### 2.2. Determination of Total Phenolic Content

The total polyphenol content of each juice (TPC-J) was determined according to the Folin–Ciocalteu (F–C) method [17]. In a 96-well microplate, 20 µL of an appropriate juice dilution or standard solution (0–50 mg/L gallic acid) was mixed with 100 µL of 10% F–C reagent. After a 3-min incubation, 80 µL Na_2_CO_3_ solution (7.5% weight/volume) was added. The plate was then incubated at 30 °C for 1 h. The absorbance at 750 nm was measured with a microplate reader (Multiskan Ascent, Thermo Labsystems, Helsinki, Finland). Results are expressed in µg gallic acid equivalents/mL juice (µg GAE/mL, GAE: gallic acid equivalents).

### 2.3. Specific Flavonoid Contents

Separation and measurement of flavonols and anthocyanins were done by Ultra-Performance Liquid Chromatography (UPLC, Waters Corporation, Milford, MA, USA), according to protocols previously described by us [18]. The total flavonol content, expressed in µg/mL, was the sum of the quercetin, myricetin, and kaempferol contents. The total anthocyanin content, expressed in µg/mL, was the sum of D, DG, DR, Cy, CyG, CyR, PG, M, MG, Pet, Pel, and P. For flavanols, we used an HSS T3 steel cartridge (Waters), 2.1 mm × 100 mm, filled with 1.8 µm particles and kept at 40 °C. The mobile phase (flow rate: 0.2 mL/min) consisted of water/acetonitrile/formic acid 91/4/5 (*v*/*v*/*v*) at time 0 and 75/20/5 after 4 min, followed by a steady state for 2 min. For GC, ECG, EGC, and EGCG, the absorbance was recorded at 280 nm. C and EC were analyzed by fluorescence spectroscopy (recording at 310 nm after an excitation at 280 nm). The total flavanol content, expressed in µg/mL, was the sum of C, EC, GC, ECG, EGC, and EGCG.

### 2.4. Vascular Reactivity

**Figure 1:**
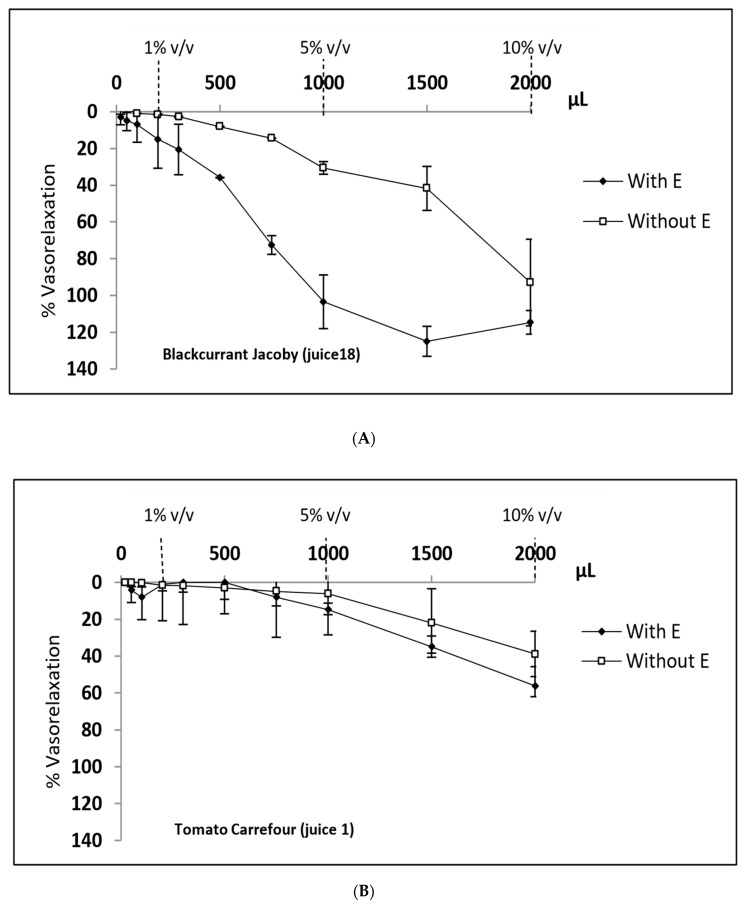
Examples of endothelial vasorelaxation activity in segments of rat aorta according to the volume of either blackcurrant (Jacoby) and Tomato (Carrefour) juices (respectively panels **A** and **B**) added to 20 mL initial organ bath solution (OBS). The following V_juice_/V_OBS_ ratios were selected to evaluate the vasorelaxant effect as a function of total or individual concentrations of specific phenolic compounds: 1%, 5%, and 10%. Cumulative volumes of each fruit or vegetable juice were then added to reach a final volume of 2000 µL over a period of 1 h. E = endothelium.

Six-week-old male Wistar rats were obtained from the Central Animal Facility of the University Hospital Center of Liège and handled as recommended by the Ethics Committee for Animal Use of the University of Liege, Belgium (file 857, accepted in 2016). After anesthesia (pentobarbital, 60 mg/mL/kg), the thoracic aorta was removed carefully, cleaned of adhering fat and connective tissue, and cut into rings (2–3 mm long). The rings were then mounted and immersed in a 20 mL organ bath solution (OBS) at 40 °C, consisting of Krebs liquid (118 mM NaCl, 25 mM NaHCO_3_, 5.5 mM d-glucose, 4.7 mM KCl, 1.18 mM KH_2_PO_4_, 2.4 mM MgSO_4_, and 3.3 mM CaCl_2_, pH 7.8) continuously bubbled with 95% O_2_ and 5% CO_2_. The rings were equilibrated for 2 h before initiating the experimental protocols. To test the reactivity of the endothelium, contraction was induced with KCl (80 mM). When contraction reached a plateau, the OB chamber was washed thrice with Krebs solution. The functional endothelium was then tested by adding first 0.5% noradrenaline (to 0.1 µM), to induce contraction, and then acetylcholine (to 10 µM), to induce at least 80% vasorelaxation. After equilibration, the rings were contracted with phenylephrine (0.5 µM) until contraction reached a plateau. Cumulative volumes of a fruit or vegetable juice (V_juice_) were then added in OBS (V_OBS_) to reach a final volume of 2000 µL (V_juice_/V_OBS_ 10%) over a period of 1 h and a concentration-relaxation curve was constructed. Three to four independent assays were run for each concentration. Figure 1 depicts typical vasorelaxation graphs obtained with juices 1 and 18 (Tomato Carrefour and Blackcurrant Jacoby). Four-parameter sigmoidal dose–response curves were fitted to the data with GraphPad Prism (version 6.0, GraphPad Software, Inc., San Diego, CA, USA). E^+^ represents the mean percentage of vasorelaxation determined at three different ratios (expressed as percentages) of juice volume to initial volume of OB solution (V_juice_/V_OBS_ ratios): 1%, 5%, and 10%. Relaxation of 100% was considered as high interest. The whole protocol was then repeated after careful removal of the endothelium to determine vasorelaxation in absence of endothelium (E^−^).

### 2.5. Statistical Analyses 

Correlations between E^+^ and TPC-J (expressed in µg GAE/mL), total polyphenols in the juice-containing OB (TPC-OB, expressed in µg GAE/mL), and total and individual flavonols, flavanols, and anthocyanins in the juice-containing OB (expressed in µg/mL) were calculated with Sisvar 5.6 software. Pearson correlations were considered significant at *p* < 0.05.

## 3. Results

**Figure 2:**
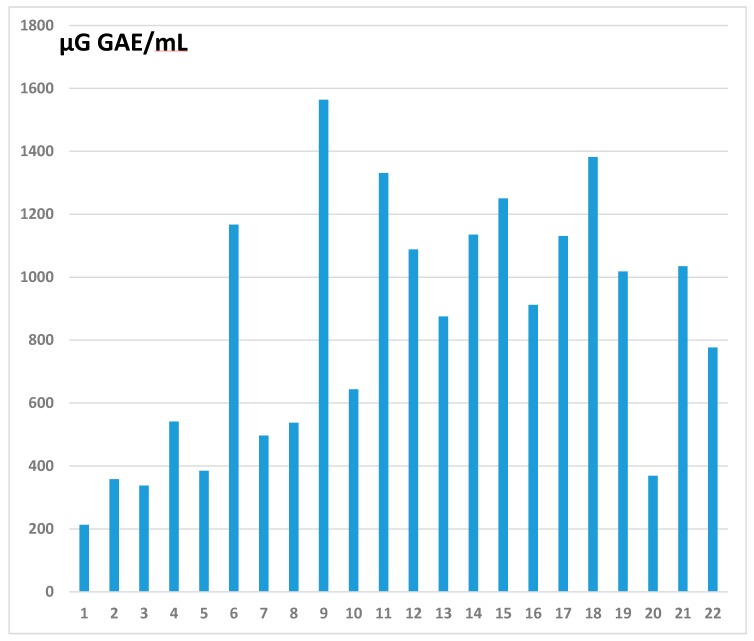
Total polyphenol contents (TPC-J), expressed in µg gallic acid equivalents per milliliter (µg GAE/mL), of 22 commercial vegetable and fruit juices found in Belgian and French markets. GAE: gallic acid equivalents. 1: Tomato (Carrefour), 2: Tomato (Biotta), 3: Carrot (Biotta), 4: Orange d’Espagne (Carrefour), 5: Pure Orange (Vitamont), 6: Lemon (Bonneterre), 7: Grapefruit, 8: Pure Grapefruit (Vitamont), 9: Grape (Materne), 10: Pure Grape (Vitamont), 11: Pomegranate (Biotta), 12: Blackcurrant (Biotta), 13: Blackcurrant (Natreen), 14: Blackcurrant (Jacoby Bio), 15: Blackcurrant (Van Nahmen), 16: Blackcurrant (Schörl Nectar), 17: Blackcurrant (Gut and Günstig), 18: Blackcurrant (Jacoby), 19: Pineapple Juice (Carrefour), 20: Pineapple Juice (De Drie Wilgen), 21: Apple (Carrefour), and 22: Pure Apple (Vitamont).

As shown in Figure 2, the range of TPC-J values was wide. Juice 1 (Tomato, Carrefour) showed the lowest value (214 µg GAE/mL) and juice 9 (Grape, Materne), the highest (1564 µg GAE/mL). The mean TPC-J for the juices investigated was 843 ± 396 µg GAE/mL. The juices also differed greatly in total flavonol and total flavanol contents, as shown in Table 1. Juice 3 (Carrot, Biotta) had the lowest flavonol content (0.8 µg/mL) and juice 12 (Blackcurrant Biotta), the highest (17.7 µg/mL). For flavanols, the lowest (2.62 µg/mL) and highest values (236 µg/mL) were observed, respectively, for juice 6 (Lemon, Bonneterre) and juice 18 (Blackcurrant, Jacoby). The average flavonol and flavanol concentrations were respectively 6.3 ± 4.7 and 109 ± 249 µg/mL. If one excludes juice 11, which reached 1194 µg/mL, the average flavanol concentration was 57.7 ± 58.2 µg/mL.

**Figure 3:**
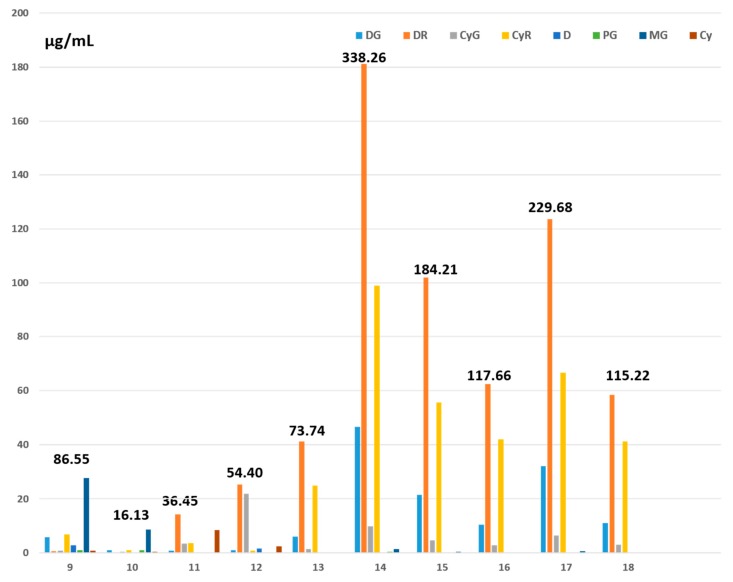
Concentrations of individual anthocyanins (µg/mL) found in 10 commercial red fruit juices. Numbers in bold indicate total anthocyanin concentrations (µg/mL) in the juices. Pet, Pel, P, and M are not represented because of their low to zero concentrations in the juices. 9. Grape Materne (Carrefour), 10. Pure Grape (Vitamont), 11. Pomegranate (Biotta), 12. Blackcurrant (Biotta), 13. Blackcurrant (Natreen), 14. Blackcurrant (Jacoby Bio), 15. Blackcurrant (Van Nahmen), 16. Blackcurrant (Schörl Nectar), 17. Blackcurrant (Gut and Günstig), and 18. Blackcurrant (Jacoby).

Among the 22 tested juices, only the 10 red ones (blackcurrant, grape, pomegranate) contained detectable levels of anthocyanins (Figure 3). Among these, the total anthocyanin content varied widely, from 16 µg/mL (Pure Grape, Vitamont) to 338 µg/mL (Blackcurrant, Jacoby Bio). We calculated mean total anthocyanin contents of 125 ± 99 µg/mL for the 10 red juices and 159 ± 99 µg/mL for the seven blackcurrant juices considered alone.

Table 2 shows the percentages of vasorelaxation E^+^ and E^-^ elicited by the 22 individual fruit and vegetable juices at three V_juice_/V_OBS_ ratios (henceforth called “volume ratios”): 1%, 5%, and 10%. Juices issued from vegetables (tomato and carrot) gave rise to a very low E^+^ when added at volume ratio 1% or 5%. At volume ratio 10%, however, the E^+^ reached 56% for juice Tomato, Carrefour, which caused similar vasorelaxation (38.83%) even after removal of the endothelium. All but one of the citrus juices exerted no vasorelaxant effect at volume ratio 1%, whether the endothelium was present or not. The exception was lemon juice, which elicited a very high percentage of vasorelaxation (81.07%) at this volume ratio, in the presence and absence of endothelium. At volume ratio 10%, the vasorelaxation caused by the other citrus juices increased up to 61.7–120%, both in the presence and absence of endothelium. Among the berry juices, those issued from blackcurrant clearly exerted the greatest vasorelaxant effect. At volume ratio 1%, all of these except juices 13 and 14 gave rise to a substantial E^+^_,_ the highest values being observed with juices 12 (44.75%) and 18 (56.76%). No significant vasorelaxant effect was observed in the absence of endothelium. At volume ratio 5%, all the blackcurrant juices elicited an E^+^ close or equal to 100%, but vasorelaxant effects were also evidenced without endothelium, albeit to a lesser extent: 41 to 67%. At volume ratio 10%, each blackcurrant juice gave rise to both E^+^ and E^-^ values equal or superior to 100%.

From the data in Figure 2 and Figure 3 and Table 1, it was possible to calculate for each OB the concentrations (in µg/mL) of total polyphenols (TPC-OB) and total and individual flavonols, flavanols, and anthocyanins and to establish correlations suggesting which compounds might be responsible for observed vasorelaxant effects. As depicted in Table 3, TPC-OB correlated significantly with E^+^ at volume ratios 5% (*r* = 0.58, *p* = 0.04) and 10% (*r* = 0.55, *p* = 0.007). No correlation was found between E^+^ and total flavonols or flavanols present in the OB. A strong, significant correlation was found between E^+^ and total anthocyanins issued from berry juices added at volume ratio 10% (*r* = 0.75, *p* = 0.05). For the flavonol family, the only correlation evidenced was between kaempferol and the E^+^ at ratio 1% (*r* = 0.56, *p* = 0.009). For the flavanol compounds, the only correlation observed was between EGCG and the E^+^ at volume ratios 1% (*r* = 0.41, *p* = 0.05) and 5% (*r* = 0.45, *p* = 0.03). For the anthocyanin family, no correlation was evidenced at ratio 1% between the E^+^ and the concentration of any individual compound in the OB. At ratio 5%, MG and especially PG from red juices (especially blackcurrant) correlated significantly with the E^+^ (respectively *r* = 0.8, *p* < 0.05 and *r* = 0.96, *p* < 0.00001). At ratio 10%, the concentrations of DG, DR, and CyR correlated significantly with the E^+^.

As shown on Table 4, the mass concentrations of phenolic compounds in each OB were converted to molar concentrations. For each volume ratio, these concentrations were averaged over all the juice-containing organ baths. The results show that an E+ was observed at all three ratios at average quercetin, kaempferol, ECG, C, EC, CyG, and PG concentrations below 1 µM. This was also the case for myricetin but only at ratios 1% and 5%. The average concentrations of EGC, EGCG, GC, DG, DR, CyR, and MG were above 1 µM.

## 4. Discussion

Many studies have evidenced that the intake of fruits and vegetables exerts potent protection against cardiovascular diseases and cancers [19,20,21,22]. All these effects have been attributed to the presence, in these foods, of antioxidants, namely polyphenols. Great attention has been paid to the cardioprotective effect of polyphenols, linked to their ability to prevent endothelial dysfunction thanks to restoration of vasodilator nitric oxide (NO) production through expression of endothelial nitric oxide synthase (eNOS) or through activation of endothelium hyperpolarization [23]. However, whether a similar link exists between cardioprotection and vegetable or fruit juice consumption is not so clear, notably because juices contain less fiber than fruits and vegetables. In 2005, Ruxton et al. [24] published a review of epidemiological and small clinical studies showing only a minor impact of fruit and vegetable juices on cancers, as compared to their greater impact on cardiovascular diseases. More recently, Hyson [25] confirmed these cancer data in a critical analysis of the scientific literature related to fruit juices and human health. The same author, however, highlighted that interventional studies with acute or chronic intake of different juices (apple, orange, mandarin, grape, cranberry, pomegranate) resulted in decreased levels of some oxidative stress biomarkers associated with increased cardiovascular risk (lipid peroxides, oxidized LDL, carbonyl groups). Khan et al. [26] have described similar observations with polyphenol-rich blackcurrant juice. To better understand the potential cardioprotective effect of fruit and vegetable juices, it is thus necessary to examine in detail how they differ in polyphenol content and composition. Surprisingly, little information is available on this topic.

Among our 22 tested juices, those prepared from lemon (juice 6), pineapple (juice 19), apple (juice 21), and especially red fruits (juices 9 to 18) clearly exhibited the highest TPC-J values. Using home-made preparations, Tzulker et al. [27] found antioxidant activity, as evidenced in DPPH assays, to correlate significantly with the TPC-J in 29 different pomegranate accessions. They gave further details about levels of total anthocyanins and four major hydrolyzable tannins. Using HPLC-PDA-MS (high performance liquid chromatography–photo diode array–mass spectrometry), Borges et al. [28] later analyzed the TPC-J values of 26 commercial pomegranate juices produced in Europe, identifying ellagitannins as the major antioxidants in these juices. Nowak et al. [29] compared the TPC-J values of five organic juices extracted from elderberry, pomegranate, cranberry, and chokeberry (*n* = 2). They observed the highest TPC-J in chokeberry juice, the predominant polyphenols being anthocyanins and phenolic acids. Granato et al. [30] examined the TPC-J of 20 organic fruit juices (cranberry, pomegranate, blueberry, elderberry, apple, and orange) acquired in local shops in the Netherlands. The pomegranate and elderberry juices exhibited the highest TPC-J (respective mean values: 2684 and 3521 µg GAE/mL). Apple and orange juices were found, respectively, to contain 399 and 470 µg GAE/mL. Wern et al. [31] report a TPC-J of 133 µg GAE/mL for fresh pomegranate juice purchased from local markets or hypermarkets in Kuala Lumpur, Malaysia, as compared to values between 3000 and 4070 mg GAE/100 mL for juices from cultivars in Spain [32] and 3150–7430 mg GAE/100 mL for juices from Chinese cultivars [33]. Here we find our selected pomegranate juice of Spanish origin to contain 1331 µg GAE/mL. As compared to the results obtained by Granato et al. [30], the TPC-J values of our orange juices (Table 1) are similar but those of our two tested apple juices are higher TPC-J (1035 and 776 µg GAE/mL). Auger et al. [34] found a TPC-J ranging from 0.31 to 1860 µg GAE/mL in 51 commercial fruit juices (grape (*n* = 12), blackcurrant (*n* = 7), cranberry (*n* = 5), apple (*n* = 6), orange (*n* = 5), red fruit (*n* = 6), blend of red fruits (*n* = 6), and non-red fruits (*n* = 4)). Among all these juices, the highest TPC-J was observed in those extracted from blackcurrant. Among the ten red juices investigated in our study, seven are blackcurrant juices. As shown in Table 1, we have found the TPC-J to vary among blackcurrant juices (from 875 to 1382 µg GAE/mL). We have calculated a mean value of 1110 ± 177 µg GAE/mL, in relatively good agreement with those observed by Lugasi and Hovari (909–1228 µg GAE/mL) [35], Auger et al. (1421 ± 222 µg GAE/mL) [34], and Tabart et al. (1530 ± 453 µg GAE/mL) [18]. Jakobek et al. [36] mention a higher TPC-J (2770 ± 64 µg GAE/mL), but for a blackcurrant juice freshly prepared with a juice extractor. Rechner et al. [37] measured a TPC-J of 3627 µg GAE/mL, but this concerned a concentrated blackcurrant juice, while commercial juices are diluted to around 30% (GlaxoSmithKline, Coleford, UK). All these differences in juice TPCs can be explained by the type of fruit or vegetable, the culture mode, and especially the processing method (milling, pressing, pasteurization, filtration, clarification, concentration), which causes more or less degradation.

Regarding polyphenol subclasses, we have found molecules of the flavonol family in all the investigated juices, their total concentration being higher than 10 µg/mL in juices 9 (Grape, Materne) and 10 (Pure Grape, Vitamont), 11 (Pomegranate, Biotta), 12 (Blackcurrant, Biotta), and 19 (Pineapple, Carrefour) (Table 2). In their concentrated blackcurrant juice, Rechner et al. [37] report myricetin, quercetin, and kaempferol concentrations of 24.9, 5.54, and 1 µg/mL, respectively, in agreement with our values for the last two compounds in the seven selected blackcurrant juices. We have also detected flavanols in all the tested juices, the total flavanol content being as high as 1194 µg/mL in juice 11 (Pomegranate, Biotta) (Table 2). By comparison, Diaz-Mula et al. [38] report a value of only 147 µg/mL in commercial pomegranate juices from the Mollar cultivar. As expected, we have found anthocyanins only in berry juices, with particularly high levels in blackcurrant juices, despite considerable disparity (54.40 to 338 µg/mL). The mean value (159 ± 99 µg/mL) found here for blackcurrant juices is in relatively good agreement with those observed by Tabart et al. [18] (228 ± 107 µg/mL). Mattila et al. [39] found a 14-fold variation in the anthocyanin content of the 12 analysed European commercial blackcurrant juice products from 17.2 µg/mL (United Kingdom) to 232 µg/mL (Germany) expressed on a ready-to-drink beverage basis. Jacobek at al [36] mention a value of 1543 ± 5.5 µg/mL in their fresh preparation, while Rechner at al [37] report a total anthocyanin content of 3118 µg/mL in their concentrated blackcurrant juice. Generally speaking, compounds of the flavonol, flavanol, and anthocyanin families represent respectively 0.7, 12, and 11–14.3% of the TPC-J. By comparison, total anthocyanins represented 55% of the TPC of the blackcurrant juice prepared with a domestic extractor and 86% of the TPC of the blackcurrant juice concentrate.

Only a few studies have examined in detail the relationship between the TPC-J and the phenolic profiles of vegetable and fruit juices and/or their potential effects ex vivo on endothelial function. To our knowledge, the most advanced paper in this field was produced by Auger et al. [34], who tested 51 commercial fruit juices and found only berry products characterized by a red-blue color (blackcurrant, grape, blueberry, cranberry, aronia) to induce marked endothelium-dependent vasorelaxation of isolated pig coronary arteries. These authors concluded, but did not formally prove, that the vasorelaxant effect of juices (E^+^ at volume ratio 1%) depends on their polyphenol composition rather than on their total polyphenol content. In a previous study [18], we confirmed in the same animal model the absence of correlation between the TPC-J and the capacity of blackcurrant juices to induce vasorelaxation. In our rat aorta model (Table 3), we have observed no correlation between E^+^ and TPC-J at the volume ratio used by Auger et al. [34]. At volume ratios 5% and 10%, in contrast, we have evidenced significant correlations: 0.58 (*p* = 0.04) and 0.55 (*p* = 0.007), respectively. The results in Table 3 lead to similar conclusions regarding the TPC-OB, use of this value being a more realistic approach.

Several previously published studies by our group as well as by others on the effect of polyphenol-rich products on the vascular system have indicated that they can induce endothelium-dependent relaxations in isolated arteries, and vasodilatation in vivo in several experimental models and in humans mainly due to the activation of both NO and endothelium-dependent hyperpolarization (EDH) pathways. Further characterization of the transduction pathways indicated that the activation of the NO pathway in response to polyphenol-rich products (red wine extract, blackcurrant, aronia, green tea, etc.) is due, at least in part, to the redox-sensitive activation of the Src/Akt/PI3-kinase pathway [23].Regarding the endothelium-independent relaxations, natural products have been shown to induce endothelium-dependent relaxations through several mechanisms including interaction with the calcium signaling pathway and an activation of the cyclic AMP and cyclic GMP relaxing pathways, in part, due to phosphodiesterase (PDEs) inhibition [40].

In our study of 22 vegetable and fruit juices, we confirm very contrasting ex vivo vasorelaxant effects between juice categories and even between juices of the same category (Table 2). In our model, the vasorelaxant activities of vegetable and citrus juices (Lemon, Bonneterre) do not appear endothelium dependent, as evidenced by similar E^+^ and E^−^ values at all volume ratios. In contrast, and in accordance with previous findings of ours [18], we find blackcurrant juices, known for their high polyphenol content, to induce substantial vasorelaxation at volume ratio 1%, in the presence but not in the absence of endothelium (exceptions: juices 13 and 14). Yet this observation appears to be influenced by the experimental conditions, since at volume ratio 10%, vasorelaxation percentages could reach 100% whether endothelium was present or not.

Of great interest is the question: among the phenolic compounds present in vegetable and fruit juices, which are the ones that favor vasorelaxation? The data in Table 3 reveal no correlation, at any volume ratio, between E^+^ and total flavonols or flavanols (mass concentrations) in the OB. At ratio 1%, however, strong correlations appear between E+ and OB concentrations of kaempferol (*r* = 0.56, *p* = 0.009) and EGCG (*r* = 0.41, *p* = 0.05). This was particularly surprising for kaempferol, given its presence in very small amount (0.1–1.2 µg/mL) in all the tested juices (Table 1). Total anthocyanins in OB, particularly those of blackcurrant juices, correlated strongly (*r* = 0.75, *p* = 0.05) with E^+^ at volume ratio 10%. Amounts of DG, DR, and CyR also correlated with E^+^ at this volume ratio. MG and especially PG (r = 0.96, P = 0.00001) correlated strongly with E^+^_,_ but at volume ratio 5%. At volume ratio 1% we found no correlation. This last observation on our rat model contrasts with our previous findings on porcine artery rings [18]. In the latter case, the potency of blackcurrant juices to induce vasorelaxation at volume ratio 1% correlated significantly with their total anthocyanin content and with their DG, DR, CyR, and MG contents. Such differences in vascular reactivity according to the volume ratio might also be related to human data indicating that flavonoid bioactivity, as reflected by in vivo endothelial function, does not follow a classical linear dose–response curve [41]. It is also possible that the rat aorta and porcine coronary artery differ markedly as regards their vascular reactivity.

What is the in vivo relevance of ex vivo data on vasorelaxation induced by phenolic compounds present in vegetable and fruit juices? This is an important question. Papers describing consistent data related to vasorelaxation induced by pure compounds are scarce. Mahobiya et al. [42] found kaempferol to induce concentration-dependent relaxation in rat pulmonary artery rings pre-contracted with phenylephrine, when present at 0.01 µM (0% vasorelaxation) to 10 µM (100% vasorelaxation). Xu et al. [43] likewise evidenced that kaempferol at 3 µM can significantly enhance bradykinin-induced relaxation of porcine coronary artery rings, through both endothelium-derived NO production and endothelium-dependent hyperpolarization. Aggio et al. [44] report 13% to 50% vasorelaxation of rat aorta in response to 1 to 100 µM EGCG. Thilavech et al. [45] report that 25 µM cyanidin-3-*O*-rutinoside (CyR) can induce 50% vasorelaxation of rat aorta. In vivo, investigators found plasma concentrations of phenolic compounds to be very low in fasted volunteers, notably because of low bioavailability. When compared to a baseline diet, human plasma quercetin concentration increased form 0.078 µM up to 0.304 µM after consumption of 110 mg/day flavonol diet during 14 days. [46]. Manach et al. [16] showed the human plasma C concentration to range between 0.14 and 0.49 µM after ingestion of 0.36 mg/kg pure C. Giordano et al. [47] report a plasma cyanidin-3-*O*-glucoside (CyG) level reaching 0.0040 µM 1h after ingestion of 500 mL orange juice containing 12.89 mg CyG. Liu et al. [48] likewise found the plasma concentration of cyanidin-3-*O*-galactoside to peak at 0.0031 µM around 2 h after ingestion of 100 g Saskatoon berries containing 123.5 mg of this compound. Rechner et al. [37] observed peak plasma concentrations of delphinidin-3-*O*-glucoside (0.006 µM), delphinidin-3-*O*-rutinoside (0.051 µM), cyanidin-3-*O*-glucoside (0.0035 µM), and cyanidin-3-*O*-rutinoside (0.024 µM) about 1 h after ingestion of 330 mL blackcurrant juice concentrate containing 1 g total anthocyanins. At least, Kay et al. [49] reported that intake of 20 g chokeberry extract containing cyanidin-3-glycosides as high as 1.3 g resulted in an average peak plasma concentration in total anthocyanins and anthocyanins metabolites of 5.1 µM within 2 h post-consumption.

After conversion of mass concentrations to molarities (Table 4), our in vitro study shows that the quercetin concentration (0.07 to 0.66 µM) able to induce vaso-relaxation in OBS at the three V_juice_/V_OBS_ ratios was in the physiological range according to data above. Unfortunately, any correlation was found between its concentration in OBS and E^+^ (Table 3). This was not the case for kaempferol whose concentration was close to or less than 0.1 µM in the vasorelaxation-inducing organ bath and significantly correlated with the E^+^ (Table 3). With respect to the flavanol family, all compounds exhibited at V_juice_/V_OBS_ 1% vasorelaxant activity below1 µM concentration in agreement with in vivo data of Manach [16]. However, only EGCG concentration correlated with E^+^ at V_juice_/V_OBS_ 1% and 5% (Table 3). Concentrations of DG, DR and CyR from the anthocyanin family correlated with E^+^ but at a level largely higher than those found in plasma. Of interest was the very strong and significant correlation (*r* = 0.96, *p* < 0.00001) found between PG at a concentration as low as 0.04 µM in OBS and E^+^ at V_juice_/V_OBS_ 5%.

Our study highlights some phenolic compounds (kaempferol, EGCG, DG, DR, CyR and PG) in relation to others as to their potential capacity to induce vascular activity. However, concentrations of DR, CyG and in a less extent of PG required for inducing ex vivo vasorelaxation remain, although largely higher than those detected in plasma. Such a difference might be explained, at least in part, by the fact that the bioavailability of phenolic compounds is often assessed in protein free-plasma, and, hence, is unable to detect natural molecules carried by proteins in blood and/or at the cell membrane. Hence, it might not be relevant to compare our data with plasma values since we have observed in preliminary experiments on rats that anthocyanins can rapidly accumulate into endothelial cells and, hence, most likely can reach much higher levels in intracellular cell compartments than in plasma. However, we also have to keep in mind the important hormetic effect of polyphenols, since they are able, at low concentrations, to stimulate the antioxydant Keap1/Nrf2/ARE system (including e-NOS) through moderate production of ROS [8,50]. Whatever the mechanism, endothelium-dependent vasodilation in both experimental models and humans has been observed after the intake of anthocyanin-rich fruit juices, indicating the ability of natural products to reach the endothelium [23]. Recently, we have shown that the intake of 400 mL of blackcurrant juice Biotta (juice 12) containing 536 mg of polyphenols (including 21.76 mg of total anthocyanins) resulted 1 h after ingestion in a significant increase (35.8%) of human endothelial function as evaluated by measurement of the Reactive Hyperemia Index (RIH) [51].

## 5. Conclusions

As pertinently noted by Habauzit and Morand [15], only a few publications report in vitro studies using polyphenols at concentrations (µM) nutritionally achievable in aglycone or conjugated form after consumption of polyphenol-containing foods. In the present paper, we have tried to show how to adequately manage and interpret information about vasorelaxation effects induced by vegetable and fruit juices under ex-vivo experimental conditions. In addition to determining TPC-J values, our first step was to assay total subclass (anthocyanins, flavanols, flavonols) and individual levels of polyphenols in the juices studied. High priority was also given to analyzing relationships between vasorelaxant intensity and total and individual phenolic compound concentrations in the organ baths rather than the juices. This enabled us to highlight that kaempferol, EGCG and peonidin-3-*O*-glucoside (PG) present in fruit juices, and more particularly the blackcurrant ones, might have some in vivo relevance, as their OB concentrations were compatible with physiological levels and correlated strongly and significantly with the E+. Yet great caution is required in interpreting data which seem to depend on experimental conditions. A simple example is the non-correlation between TPC-J or TPC-OB and vasorelaxant intensity at V_juice_/V_OBS_ ratio 1%, in contrast to the correlations observed at 5% or 10%.

Even if endothelium-dependent vasodilation in both experimental models and humans have been reported after the intake of anthocyanin-rich fruit juices [23], large-scale clinical studies are needed to further and better explore in humans the beneficial endothelium-dependent cardiovascular effects of vegetable and fruit juice intake [52] and hence of polyphenols present in these foods [41]. This requires evaluating in vivo endothelial function in three with the measurement of plasma concentrations of both polyphenol metabolites by HPLC-MS/MS [48] and NO. On the basis of results from such an effective battery of tests, the European Food Safety Authority has authorized, under article 13(5) of Regulation (EC) No 1924/2006, a health claim related to cocoa flavanols and maintenance of normal endothelium-dependent vasodilation. By contrast, the PRISMA-compliant meta-analysis recently performed by Zhu et al. [53] does not favor any blood pressure-improving clinical efficacy of anthocyanin supplementation.

## Figures and Tables

**Table 1:** Individual and total flavonol and flavanol contents (µg/mL) of 22 commercial vegetable and fruit juices.

number	List of Juices	Myricetin	Quercetin	Kaempferol	Total Flavonols	EGC	EGCG	ECG	GC	C	EC	Total Flavanols
1	Tomato (Carrefour)	0.3	2.7	0.1	3.1	2.74	8.86	1.64	53.55	0.05	4.34	71.19
2	Tomato (Biotta)	0.1	1.6	0.2	1.9	14.40	8.34	3.89	26.70	0.06	2.67	56.06
3	Carrot (Biotta)	0.1	0.5	0.2	0.8	0.71	29.64	1.58	0.00	0.32	0.85	33.09
4	Orange d’Espagne (Carrefour)	0.5	0.9	0.1	1.5	20.47	7.91	1.57	0.60	0.21	0.22	30.97
5	Pure Orange (Vitamont)	1.3	0.8	0.7	2.8	7.69	2.67	2.19	1.53	0.09	1.09	15.25
6	Lemon (Bonneterre)	0.7	1.3	1.9	4.0	0.52	0.86	0.42	0.11	0.03	0.68	2.62
7	Grapefruit	1.8	2.0	1.1	4.9	4.18	1.48	0.85	0.24	0.14	3.15	10.03
8	Pure Grapefruit (Vitamont)	0.6	0.9	0.5	2.0	9.67	1.99	1.38	0.03	0.04	4.66	17.76
9	Grape Materne (Materne)	13.0	2.7	0.4	16.0	86.68	41.37	14.43	3.89	1.11	7.51	154.99
10	Pure Grape (Vitamont)	7.6	3.3	0.9	11.9	5.63	11.79	8.85	0.27	0.36	1.81	28.71
11	Pomegranate (Biotta)	8.5	4.0	0.2	12.7	849.59	45.95	3.00	294.60	0.09	0.77	1194
12	Blackcurrant (Biotta)	14.4	2.5	0.7	17.7	13.85	37.80	7.11	2.00	0.02	0.63	61.41
13	Blackcurrant (Natreen)	4.8	3.5	0.0	8.3	23.00	18.42	2.10	1.20	0.10	1.39	46.22
14	Blackcurrant (Jacoby Bio)	1.6	2.2	0.3	4.1	21.81	33.17	1.73	4.29	0.26	2.17	63.43
15	Blackcurrant (Van Nahmen)	1.8	2.7	0.5	4.9	22.60	66.02	2.78	10.76	0.33	1.71	104.20
16	Blackcurrant (Schlör nectar)	2.0	1.9	0.3	4.2	22.68	41.27	1.96	6.41	0.22	0.84	73.39
17	Blackcurrant (Gut and Günstig)	1.9	2.7	0.6	5.1	21.90	95.06	4.46	7.77	0.35	2.31	131.85
18	Blackcurrant (Jacoby)	3.2	3.3	0.7	7.2	44.05	181.42	3.25	4.65	0.60	2.07	236.02
19	Pineapple juice (Carrefour)	7.7	1.6	1.2	10.5	1.74	4.66	1.12	0.32	0.12	3.59	11.55
20	Pineapple juice (De Drie Wilgen)	1.6	0.9	0.4	2.9	8.32	14.06	4.94	18.94	0.02	2.40	48.67
21	Apple (Carrefour)	1.3	2.3	1.0	4.5	1.26	3.17	1.37	0.03	0.11	0.45	6.39
22	Pure Apple (Vitamont)	3.5	4.0	0.3	7.9	1.32	2.87	2.80	0.04	0.14	0.74	7.91

**Table 2:** Ability of 22 commercial vegetable and fruit juices to induce vasorelaxation in segments of rat aorta. E^+^ and E^-^ represent percentages of vasorelaxation observed at three V_juice_/V_OBS_ ratios (1%, 5%, and 10%), respectively in the presence (+) and absence (−) of endothelium.

Number	Juices Origin	With Endothelium			Without Endothelium		
		E^+^ (1% *v*/*v*)	E^+^ (5 % *v*/*v*)	E^+^ (10 % *v*/*v*)	E^−^ (1% *v*/*v*)	E^−^ (5% *v*/*v*)	E^−^ (10% *v*/*v*)
1	Tomato (Carrefour)	1.19 ± 22.87	14.73 ± 3.44	56.19 ± 10.44	1.51 ± 3.1	6.07 ± 11.49	38.83 ± 12.3
2	Tomato (Biotta)		6.9 ± 7.73	28.82 ± 9.63		30.58 ± 5.85	79.82 ± 7.50
3	Carrot (Biotta)	4.28 ± 6.7	7.72 ± 6.39	44.94 ± 18.26	8.52 ± 1.84	35.24 ± 9.45	63.41 ± 22.19
4	Orange d’Espagne (Carrefour)		27.86 ± 27.04	61.70 ± 30.32	6.1 ± 14.35	25.79 ± 16.05	49.68 ± 24.24
5	Pure Orange (Vitamont)		40.63 ± 30.89	83.27 ± 21.00	4.11 ± 4.75	122.9 ± 66.61	173.14 ± 89.22
6	Lemon (Bonneterre)	81.07 ± 9.9	95.06 ± 13.4	87.32 ± 10.97	79.35 ± 1.49	102.04 ± 1.25	98.48 ± 1.11
7	Grapefruit		16.64 ± 17.04	74.39 ± 1.34		36.17 ± 10.46	89.68 ± 2.70
8	Pure Grapefruit (Vitamont)	15.01 ± 0.00	106.56 ± 6.86	120.43 ± 7.10		102.49 ± 6.86	105.41 ± 7.10
9	Grape Materne (Materne)	8.81 ± 0.80	22.98 ± 5.50	45.11 ± 9.58		16.77 ± 4.51	40.49 ± 4.12
10	Pure Grape (Vitamont)	15.01 ± 0.23	20.06 ± 12.54	56.19 ±11.05	0.18 ± 17.56	25.76 ± 22.59	38.83 ± 1.92
11	Pomegranate (Biotta)		93.13 ± 8.42	106.78 ± 5.67	2.09 ± 1.91	59.35 ± 17.94	92.34 ± 19.73
12	Blackcurrant (Biotta)	44.75 ± 22.76	97.29 ± 26.8	110.15 ± 15.87	4.56 ± 7.66	40.56 ± 22.31	94.35 ± 27.06
13	Blackcurrant (Natreen)	9.47 ± 16.59	106.55 ± 4.19	114.65 ± 13.14		42.59 ± 31.06	66.56 ± 30.63
14	Blackcurrant (Jacoby Bio)	5.12± 0.92	96.59 ± 12.4	191.68 ± 3.81		44.21 ± 12.37	91.57 ± 18.88
15	Blackcurrant (Van Nahmen)	19.9 ± 0.97	100.53 ± 5.08	109.73 ± 7.52	2.76 ± 42.66	64.48 ± 25.82	127.59 ± 18.8
16	Blackcurrant (Schörl nectar)	23.75 ± 3.59	85.57 ± 20.41	117.41 ± 8.91	1.64 ± 0.14	42.07 ± 20.44	87.59 ± 26.57
17	Blackcurrant (Gut & Günstig)	19.99 ± 14.51	85.88 ± 8.10	107.18 ± 2.61		52.29 ± 9.00	110.93 ± 7.56
18	Blackcurrant (Jacoby)	56.76 ± 24.49	103.82 ± 5.07	106.1 ± 7.97	0.87 ± 1.23	67.53 ± 15.57	103.43 ± 4.16
19	Pineapple juice (Carrefour)	5.66 ± 14.96	13.04 ± 6.9	35.46 ± 2.12		9.1 ± 7.54	38.19 ± 20.4
20	Pineapple juice (De Drie Wilgen)		12.95 ± 2.19	92.05 ± 0.72		5.88 ± 1.58	34.15 ± 5.00
21	Apple (Carrefour)			29.23 ± 6.97		0.98 ± 3.23	17.83 ± 1.34
22	Pure Apple (Viamont	15.28 ± 4.75	64.01 ± 4.29	86.64 ± 11.96		32.38 ± 19.4	68.68 ± 27.3

**Table 3:** Correlations between E^+^ and TPC-J, TPC-OB, and total and individual OB concentrations of juice flavonols, flavanols, and anthocyanins, according to the volume of juice added to 20 mL initial OB solution (V_juice_/V_OBS_ ratios 1%, 5%, and 10%). E^+^ represent percentages of vasorelaxation observed at three V_juice_/V_OBS_ ratios in the presence of endothelium.

Compounds	E^+^ (1% *v*/*v*)		E^+^ (5% *v*/*v*)		E^+^ (10% *v*/*v*)	
	*r*	*p*-Value	*r*	*p*-Value	*r*	*p*-Value
TPC-J	0.32	0.14	0.58	0.04	0.55	0.007
TPC-OB	0.32	0.14	0.58	0.04	0.55	0.007
Flavonols-OB	0.19	0.39	0.17	0.44	0.02	0.99
Flavanols-OB	0.08	0.69	0.27	0.2	0.17	0.44
Anthocyanins-OB	0.54	0.2	0.28	0.5	0.75	**0.05**
Myricetin	0.1	0.6	0.06	0.7	0.07	0.7
Quercetin	0.11	0.61	0.35	0.1	0.19	0.38
Kaempferol	0.56	0.009	0.11	0.96	0.19	0.4
EGC	0.14	0.51	0.22	0.31	0.13	0.54
EGCG	0.41	0.05	0.45	0.03	0.31	0.15
ECG	0.004	0.98	0.13	0.5	0.17	0.44
GC	0.19	0.3	0.14	0.5	0.09	0.68
C	0.07	0.75	0.03	0.87	0.09	0.67
EC	0.19	0.37	0.2	0.35	0.16	0.45
DG	0.17	0.6	0.3	0.39	0.70	0.02
DR	0.06	0.85	0.49	0.14	0.81	0.004
CyG	0.38	0.26	0.37	0.28	0.39	0.25
CyR	0.06	0.86	0.44	0.2	0.76	0.01
PG	0.49	0.28	**0.96**	0.00001	0.61	0.1
MG	0.36	0.2	0.8	0.05	0.61	0.11

**Table 4:** Conversion of individual flavonol, flavanol, and anthocyanin mass concentrations to molarities (µM). The indicated values are the means ± SD calculated for the whole set of juices. * For EGC and GC, values related to juice 11 (pomegranate) were removed for the three ratios because being aberrant.

Compounds	Title		
	V_juice_/V_OBS_ 1%	V_juice_/V_OBS_ 5%	V_juice_/V_OBS_ 10%
flavonols (µM)			
myricetin	0.12 ± 0.13 (0.005–0.11)	0.56 ± 0.62 (0.022–2.60)	1.06 ± 1.18 (0.042–4.12)
quercetin	0.07 ± 0.03 (0.17–0.62)	0.34 ± 0.16 (0.080–0.623)	0.66 ± 0.31 (0.15–1.2)
kaempferol	0.02 ± 0.02 (0.006–0.67)	0.09 ± 0.07 (0.024–0.32)	0.18 ± 0.14(0.046–0.615)
flavanols (µM)			
EGC	0.51 ± 0.60 (0.02–2.8)*	2.5 ± 3.0 (0.08–6.8)*	4.8 ± 5.9 (0.21–6.8)*
EGCG	0.6 ± 0.9 (0.02–3.9)	3.10 ± 4.4 (0.09–19)	6.0 ± 8.3 (0.17–36)
ECG	0.1 ± 0.1 (0.01–0.32)	0.4 ± 0.4 (0.05–1.60)	0.7 ± 0.7 (0.09–3)
GC	0.23 ± 0.4 (0.01–1.7)*	1.1 ± (0.01–8.3)*	2.03 ± 3.8 (0.01–16)*
C	0.01 ± 0.01 (0.002–0.38)	0.04 ± 0.04 (0.004–0.18)	0.1 ± 0.1 (0.007–0.35)
EC	0.1 ± 0.1 (0.02–0.26)	0.3 ± 0.3 (0.04–1.20)	0.7 ± 0.6 (0.07–2.40)
anthocyanins (µM)			
DG	0.29 ± 0.3 (0.02-0.99)	1.39 ± 1.6 (0.08-4.76)	2.66 ± 3.0 (0.14-9.10)
DR	1.97 ± 1.9 (0.01–5.90)	9.48 ± 9.1 (0.03–28)	18.22 ± 17.6 (0.06–54)
CyG	0.12 ± 0.10 (0.01–0.48)	0.57 ± 0.7 (0.05–2.30)	1.10 ± 1.3 (0.09–4.42)
CyR	0.45 ± 0.40 (0.01–1.30)	2.15 ± 2.10 (0.05–6.20)	4.11 ± 4.0 (0.10–12)
PG	0.01 ± 0.01 (0.01–0.02)	0.04 ± 0.04 (0.02–0.11)	0.08 ± 0.07 (0.04–0.21)
MG	>10	>20	>20
